# Prospects of implant with locking plate in fixation of subtrochanteric fracture: experimental demonstration of its potential benefits on synthetic femur model with supportive hierarchical nonlinear hyperelastic finite element analysis

**DOI:** 10.1186/1475-925X-11-23

**Published:** 2012-04-30

**Authors:** Mohammed Hadi Latifi, Kunalan Ganthel, Shanmugam Rukmanikanthan, Azura Mansor, Tunku Kamarul, Mehmet Bilgen

**Affiliations:** 1 National Orthopaedic Centre of Excellence in Research and Learning, University of Malaya, 50603 Kuala Lumpur, Malaysia; 2 Department of Orthopedic Surgery, Faculty of Medicine, University of Malaya, 50603 Kuala Lumpur, Malaysia; 3 Health and Translational Medicine, Faculty of Medicine, University of Malaya, 50603 Kuala Lumpur, Malaysia

**Keywords:** Locking plate, Angle blade plate, Dynamic condylar screw plate, Subtrochanteric fracture, Biomechanics, Finite element analysis, Hierarchical finite element modelling

## Abstract

**Background:**

Effective fixation of fracture requires careful selection of a suitable implant to provide stability and durability. Implant with a feature of locking plate (LP) has been used widely for treating distal fractures in femur because of its favourable clinical outcome, but its potential in fixing proximal fractures in the subtrochancteric region has yet to be explored. Therefore, this comparative study was undertaken to demonstrate the merits of the LP implant in treating the subtrochancteric fracture by comparing its performance limits against those obtained with the more traditional implants; angle blade plate (ABP) and dynamic condylar screw plate (DCSP).

**Materials and Methods:**

Nine standard composite femurs were acquired, divided into three groups and fixed with LP (n = 3), ABP (n = 3) and DCSP (n = 3). The fracture was modeled by a 20 mm gap created at the subtrochanteric region to experimentally study the biomechanical response of each implant under both static and dynamic axial loading paradigms. To confirm the experimental findings and to understand the critical interactions at the boundaries, the synthetic femur/implant systems were numerically analyzed by constructing hierarchical finite element models with nonlinear hyperelastic properties. The predictions from the analyses were then compared against the experimental measurements to demonstrate the validity of each numeric model, and to characterize the internal load distribution in the femur and load bearing properties of each implant.

**Results:**

The average measurements indicated that the constructs with ABP, DCPS and LP respectively had overall stiffness values of 70.9, 110.2 and 131.4 N/mm, and exhibited reversible deformations of 12.4, 4.9 and 4.1 mm when the applied dynamic load was 400 N and plastic deformations of 11.3, 2.4 and 1.4 mm when the load was 1000 N. The corresponding peak cyclic loads to failure were 1100, 1167 and 1600 N. The errors between the displacements measured experimentally or predicted by the nonlinear hierarchical hyperelastic model were less than 18 %. In the implanted femur heads, the principal stresses were spatially heterogeneous for ABP and DCSP but more homogenous for LP, meaning LP had lower stress concentrations.

**Conclusion:**

When fixed with the LP implant, the synthetic femur model of the subtrochancteric fracture consistently exceeds in the key biomechanical measures of stability and durability. These capabilities suggest increased resistance to fatigue and failure, which are highly desirable features expected of functional implants and hence make the LP implant potentially a viable alternative to the conventional ABP or DCSP in the treatment of subtrochancteric femur fractures for the betterment of clinical outcome.

## Background

Subtrochanteric femur fracture (SFF) is a common occurrence and requires surgical intervention with an orthopaedic implant [1]. A stable fixation restores the weight bearing function of the bone and provides a stable environment for fracture healing. Angle blade plates (ABP) and dynamic condylar screw plate (DCSP) are the two extramedullary implants commonly used for fixing SFF. At the time of surgery, fixation with either implant may appear stable, but may eventually fail. On a group of 18 patients, the failure rate of the ABP implant was reported to be 39 % [2]. In a study with 36 patients, the rate for the DCSP implant was 17 % [3]. More recent study indicated that the failure rate varied between 20 % and-30 % within the first 12 weeks of surgery, depending on the age of the patient [4]. The early failure is largely attributed to the inability of the implants to withstand typical loading conditions experienced during normal human activities.

Locking plate (LP) is another orthopaedic implant, but used mainly for stabilizing the distal fractures of the femur near the knee with favourable long-term outcome [5]. In a study with 14 patients, the failure rate of the LP implant was reported to be 14 % [6]. Because its contour fits well to the contra lateral surface of the proximal femur, LP has also been considered as an alternative option for the fixation of SFF. Unlike ABP and DCSP, LP exploits a different strategy and employs multiple screws equipped with a special locking mechanism. Such capability prevents slippage or loosening of the screws when mounted into the head or shaft of the femur. This particular implant also leaves a small gap between the plate and bone surfaces to maintain periosteal blood supply. Also, it is surgically implanted with less invasive procedures, which lead to fast recovery. These features collectively make LP an attractive alternative over the other implants ABP and DCSP [7]. Based on its success record in fixing the distal femur fractures, we hypothesized that, when feasible, LP would also yield more robust fixation of the SFFs by supporting more uniform load distribution in bone/implant interfaces, especially in the regions surrounding the fastening points of the implant. The end configuration of the SFF fixation with LP would therefore provide more favourable mechanical environment as compared to those achieved with the conventional implants ABP and DCSP.

In this study, we tested the performances of three implants ABP, DCSP and LP with both experimental and numerical approaches. Experiments were conducted on simulated femur constructs where the implants were secured to the outer contours of the synthetic femurs by following the procedures identical to those performed in a typical SFF surgery. Then, a 20 mm long segment was cut off and removed completely from the subtrochanteric region of each sample. The created gap was intentionally made large to produce an extreme case of fracture, which enabled studying the biomechanical response of each individual implant design alone without the interference from the fracture itself or its opposing interface surfaces on different loading conditions. The sample models with implants were subjected to biomechanical tests using axial compressive loading under both static and dynamic conditions. The resulting measurements were recorded and compared for evaluating the load bearing properties and failure limits of each implant. To mechanistically understand the critical interactions at the implant boundaries and also to confirm the experimental measurements, finite element analyses of the synthetic femur/implant systems were also performed in parallel. The investigations involved constructing hierarchical finite elements with nonlinear hyperelastic properties and performing computations to characterize the internal load distribution in the femur and load bearing properties of each implant. The hierarchical modelling approach allowed assigning different shapes and material properties to the finite elements selected within the cortical and cancellous sections of the synthetic femur. The use of nonlinear hyperelasticity allowed realistically mimicking the biomechanics of the composite materials in the cortical and cancellous sections as they were measured directly from the samples that were cut off from each section. The model predictions were then compared against the experimental data to demonstrate the validity of the models and explain the experimental measurements. In the following sections, we describe each of these processes and discuss our findings in detail within the scope of whether the implant LP would offer better biomechanical performance in fixing SFF than the other two implants ABP and DCSP.

## Materials and Methods

### Synthetic femur and implants

**Figure 1:**
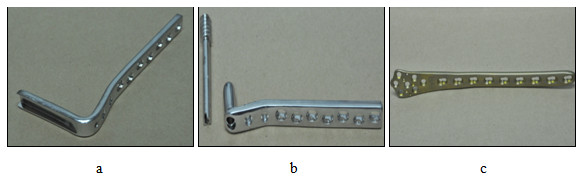
Implants used in this study: a - Angle blade plate (ABP), b - Dynamic condylar screw plate (DCSP) and c - Locking plate (LP).

Nine small size simulated femurs representing the left side were purchased from a commercial company (Sawbones, Pacific Research Laboratories, Inc., Vashon, WA, USA). The simulated femurs were constructed using a fourth generation composite technology to be nearly identical to the real human femur in terms of its biomechanical structure, function and properties [8]. The age and quality of the cadaveric bone affects its stiffness and hence yields variations in biomechanical response from one sample to the next [9]. For this reason, composite femurs are well utilized in mechanical tests and preferred over the corresponding cadaveric femur for minimizing the interspecimen variation. Three sets of implants; 130° angle blade plate (ABP) and dynamic condylar screw plate (DCSP), both made of standard stainless steel, and a locking plate (LP) implant made of titanium alloy were all purchased from the same company Synthes® with catalog numbers 238.98, 237.94 and 422.255, respectively (Synthes, Inc., Solothurn, Switzerland). The implants were shown in Figure 1 and their specifications were summarized in Table 1.

**Table 1:** Comparison of the physical specifications of the implants angle blade plate (ABP), dynamic condylar screw (DCSP) and locking plate (LP) used in this study

**Implant type**	**Number of holes**	**Femoral head fixation**	**Femoral shaft fixation**
ABP (stainless steel)	9	90 mm blade at 130° angle	Five 36 mm bicortical screws in 5 shaft holes (4.5 mm)
DCSP (stainless steel)	10	85 mm standard lag screw and 58 mm cortical screw (4.5 mm)	Five 36 mm bicortical screws in 5 shaft holes (4.5 mm)
LP (titanium)	7 on plate head and		
9 on plate shaft	Five locking screws inserted into the head (4.5 mm)	Five locking screws inserted in 5 shaft holes (4.5 mm)	

**Figure 2:**
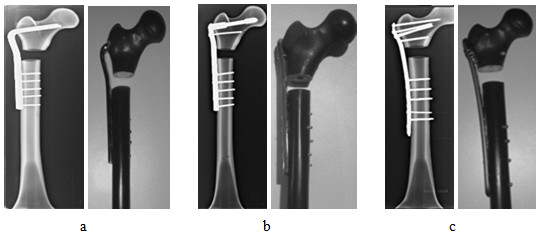
Radiographic (left) and visual (right) images of the composite constracts of SFF that were fixed with the implants a - ABP, b - DCSP and c - LP.

**Figure 3:**
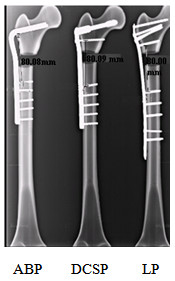
Working lengths as measured on the radiographic images of the composite constructs of SFF fixed with the three implants.

The implant was first fixed to the synthetic femur. Then, osteotomy was performed at 70 mm distal from the tip of the greater trochanter in the subtrocthanteric region. Below this region, a segmental defect measuring 20 mm in length was created, similar to the one described in [10]. The resulting medial calcar comminution was considered as the extreme representation of SFF. This arrangement however allows studying the biomechanical response of the individual implant design without the interference from the bone/fracture interaction when the fracture was only partial or not complete across the bone. Subsequently, radiographic and photographic images of the femur/implant constructs were acquired for visual display and mesh generation in finite element analysis. Figure 2 compares the constructs with three different plate designs side-by-side. The closest distance between the implant elements mounted in the proximal and distal segments of the femur was considered as working length. The working lengths were nearly identical for all implants, as depicted by the radiographic images in Figure 3.

### Mechanical testing of the constructs

**Figure 4:**
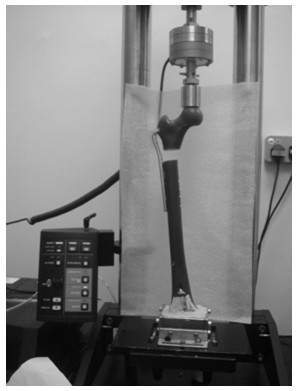
Placement of an implanted composite femur construct with SFF in an Instron machine for mechanical testing.

The femur constructs were all tested mechanically under both static and dynamic loading conditions using a materials-testing machine (Instron 5800 R, Canton, MA, USA), as shown in Figure 4. The distal femoral condyle was fixed using a dental stone (plaster based on gypsum powder mixed with water) enclosed within a custom-built jig for positioning at the base of the Instron machine. During the test, the femoral head was placed under a stainless steel jig with a concave depression, physically conforming the superior curvature of the head. With the help of a plummet, each construct was aligned vertically so that the applied compression was in line with the mechanical axis of the construct in both sagittal and coronal planes.

The static axial loading was performed under the mode of linear elastic control and involved an initial 100 N of preloading that was followed by displacement at a rate of 10 mm/min until 500 N of load was reached. At the maximum load of 500 N, the axial deformation of each construct exhibited linear elastic behaviour. Otherwise, plastic deformation would stop the operation of the Instron machine. Force and displacement measurements were read and stored by using Bluehill2® software, which was provided by the manufacturer of the Instron machine. The temporal profiles of the force and displacement measurements exhibited nearly a linear trend when plotted on the same graph, and hence fitted to a linear function using regression analysis, as demonstrated in reference [7]. The stiffness of the overall construct was estimated from the slope. The stiffness obtained from the static loading of each construct was a critical parameter and compared against the one predicted by the corresponding numerical model, which was simulated identical to the experimental conditions, as described below.

After the static loading, the same construct was subjected to dynamic axial loading test. The dynamic test protocol started similarly with a preload of 50 N and followed by a cyclic loading of 300 N applied under the displacement control mode of 1 mm/s. After reaching ten cycles, the load was incremented by 100 N without stopping, and the test was repeated with 400 N. This incremental loading paradigm with ten cycles in each phase continued until the implant was maximally deformed and that the two medial edges of the implanted sites of the femur came to contact. At this point, the amount of load was recorded as the indicator of the peak cyclic load to failure. The magnitudes of the minimum and maximum displacements during each phase of the cyclic loading were measured to determine the reversible and irreversible (plastic) deformations [11]. The reversible deformation was determined as the average difference between the distances of the distal and proximal edges of the gap before and after each cycle of the loading. The plastic deformation was another critical parameter used for determining the durability of the implants and comparing their performances. It was defined as the minimum distance, i.e., the distance from the top portion of the curve to the zero line.

### Finite element models of the constructs

**Figure 5:**
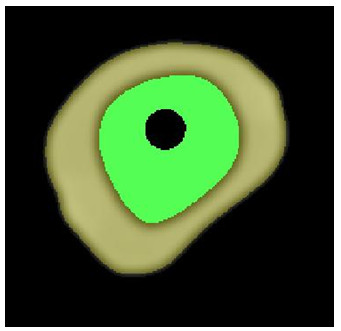
Segmentation of the the cortical (gray color) and cancellous (green color) sections on the cross sectional computer tomography image of a synthetic femur.

Finite element model (FEM) of each construct was built to numerically simulate the geometric and material properties of the femur constructs with specific implants as realistically as possible based on the underlying structural hierarchy using software ABAQUS (FEA Solver, Realistic Simulation and 3D analysis - Dassault Systèmes, Villacoublay Cedex, France). This process required information about the actual geometry describing the contours of the internal and external surfaces of the synthetic femur, the internal boundaries between its cortical and cancellous sections and the geometry of the elements of each implant. Each construct was imaged using a computer tomography scanner (As + 128 Somatom model, Siemens, Inc., Henkestrasse, Germany). The image acquisition parameters were 140 kVp; 80 mA; 1 s; slice thickness = 0.6 mm and number of slices = 757, which covered the whole length of the construct. The acquired images visualizing the synthetic femur in transverse plane were postprocessed offline to segment out its cortical and cancellous sections. The brightness and contrast of a selected image in the series were first adjusted until we could clearly differentiate the boundary between the cortical and cancellous regions. Next, a threshold value was selected manually from the spatial intensity distribution of the image and applied to segment out these two distinct regions. Figure 5 shows an example obtained by this approach. We note that the same threshold value was applied to the other remaining images of the femur simultaneously to achieve segmentation in 3 D.

**Figure 6:**
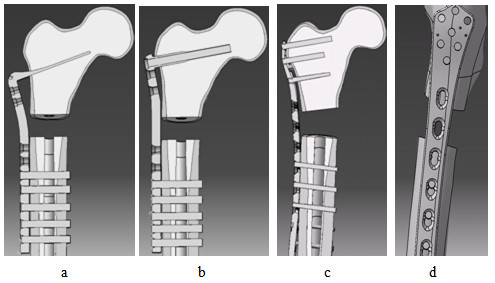
Cross sectional views of the composite constructs of SFF fixed with the implants a - ABP, b – DCSP, and c – LP. d – Side view of the implantation in c.

The plates and screws of each implant were adapted into the 3 D geometry of the femur construct. The implants were sent to a company (KL Analytical Sdn Bhd, Kuala Lumpur, Malaysia) to generate their digital representations in 3 D. The screw holes in the plates were further trimmed using software CATIA V5 (Virtual Design for Product Excellence - Dassault Systèmes, Villacoublay Cedex, France). Each screw was modelled as a filled cylinder with no treading. The screw bodies were removed from the cortical and cancellous sections according to the implant design. The plates, screws and synthetic femur were all assembled together using again CATIA V5 and imported to ABAQUS. The sharp surfaces were filleted to avoid mesh irregularities or highly distorted elements. Figure 6 shows the geometries of the final constructs given in coronal view along the midline (a-c) and the side view (d).

### Assignments of the hyperelastic material properties

Material properties of the cortical and cancellous sections of the synthetic femurs were measured experimentally according to the ASME load-stroke protocol [12]. Uniform cubic samples with 1 cm in each direction were first removed from the cortical and cancellous sections of the synthetic femur and tested using Instron machine under uniaxial compression and tensile conditions which were applied at a displacement rate of 10 mm/min. This rate is typically set as reference in characterizing the composite systems [13]. In uniaxial compression, the samples were deformed vertically using plates with flat surfaces. For tensile test, two handle bars were glued to the two opposite surfaces of the samples and attached to the fixtures of Instron machine for pulling. The resulting nominal stress–strain measurements were promptly fed into ABAQUS using its hyperelastic subdivision with Neo-Hookian as strain energy potential and uniaxial volumetric test data option. The stress–strain measurements from the cubes were also analyzed independently to determine the elastic properties of the composite materials used in the cortical and cancellous sections of the synthetic femur. Table 2 lists the results obtained experimentally as well as those reported by the manufacturer of the synthetic femur. We note that our estimates were based on the readings taken from the displacement points where the cubes were either started to break under the tensile or ruptured under the compressive loading. This meant that the estimation range covered beyond the linear region and hence included nonlinearity in the stress–strain curve. Comparing the corresponding values in the table, the inclusion of the nonlinearity may explain why our measurements yielded slightly lower values for the mechanical properties (as defined by strength and modulus parameters) under both the tensile and compressive loading conditions. Because of this difference, we opted to use the hyperelastic definition in the numerical simulations, as described above. With this choice, Poisson ratio was calculated inherently by ABAQUS from the imported data.

**Table 2:** Experimentally measured elastic properties of the cortical and cancelous sections of synthetic femur compared against those reported by the manufacturer of the synthetic femur

	**Tensile**				**Compressive**			
	Strength (MPa)		Modulus (GPa)		Strength (MPa)		Modulus (GPa)	
	EMV	SRV	EMV	SRV	EMV	SRV	EMV	SRV
Cortical	98.6	106	14.43	16.0	146.33	157	14.62	16.7
Cancellous	1.3	-	0.24	-	4.82	5.40	0.125	0.137

EMV denotes experimentally measured value. SRV denotes the value reported by the manufacturer (Sawbones, Pacific Research Laboratories, Inc., Vashon, WA, USA) of the synthetic femur.

The plates of the implants were defined by homogeneous linear elastic properties. The elastic modulus and possion ratio for the metal of each plate were determined from the literature and listed in Table 3[10]. The values in the table were accordingly assigned to the geometry of the corresponding plate in ABAQUS.

**Table 3:** Comparison of the elastic properties of the industrial metals from which ABP, DCSP and LP were manufactured

**Implant**	**Material**	**Elastic Modulus (GPa)**	**Poisson ratio ϑ**
ABP	Stainless steel	200	0.3
DCS	Stainless steel	200	0.3
LP	Titanium	114	0.33

Include the elastic properties of the composite materials used to construct the synthetic femur.

### Interactions between the implant and composite femur interfaces

Numerical modelling using ABAQUS required specifying the mechanical contact properties in the implant and composite femur system. The contact property options in the software included normal and tangential interactions between two materials with a common interface.

**Figure 7:**
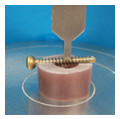
Setup for penetration test to determine the normal interaction property of the screw and composite femur.

The normal behaviour between the screw and composite femur was derived from a separate standard penetration test. In this test, a piece of composite femur was segmented out from its shaft, a screw was placed horizontally on its top and compression load was applied on the screw through the tip of the Instran machine, as shown in Figure 7. The pressure-overclosure data obtained from this test were fed into ABAQUS. In the software, the constrained enforcement method was set as standard and pressure-overclosure was set as hard contact. The contact stiffness behaviour was defined as nonlinear and maximum stiffness value was set as default. Under the default option, the software automatically fitted the data to an exponentical function describing the normal contact behaviour indicated by stiffness scale factor = 1, initial/final stiffness ratio = 0.01, upper quadratic limit scale factor = 0.03, lower quadratic limit ratio = 0.3333 and clearance at which contact pressure = 0.

The tangential behaviour between the screw and composite femur was defined as rough. This setting indicated that the two points contacting each other between the screw and femur elements would not slip.

For ABP, the normal behaviour of the interaction between the blade and femur head was described by hard contact. But, the tangential behaviour was defined with the selections: friction formulation = penalty, directionality = isotropic, friction coefficient =3.76 and shear stress limit = 200. The values for the friction coefficient and shear stress limit were determined from the pull out test, which was performed by following the procedures in [14]. The interactions in all remaining interfaces between the plates and femur shaft were defined by normal behaviour only, but with hard contact option.

For the interactions between the screws and blades of ABP and DCSP, the normal behaviour was set to hard contact and the tangential behaviour was set to frictionless. For LP, however, because of the locking mechanism on the screw heads, direct normal and rough tangential behaviours were defined between the screws and the blade.

### Boundary conditions and loading

The interface between the cortical and cancellous sections of the synthetic femur were bind together according to the ENCASTRE definition under the boundary-condition option of ABAQUS. This option allowed rigidly joining the finite elements with a common border shared by the two sections. To simulate the stable positioning of the constructs on the Instron machine, the surface nodes of the methacondylar section of the digitized femur construct was constrained so that they would not move freely. But, the other digitized external and medullar surfaces of the synthetic femur was set free, meaning that they were allowed to move freely in six degrees of freedom.

For applying static compressional force, a point source has been assigned on the finite element node at the highest altitude in the digitized femur head and its direction was set vertical. To mimic the static experimental condition, a preload of 50 N was absorbed into the force applied initially and this force was set to a magnitude of 150 N at the first step and incremented by 100 N in the following 5 steps until the final load of 550 N was reached.

Furthermore, in the software, general static steps were defined for loading increments. And step incremental size was decreased to 1x10^-8^ for faster and more accurate convergence.

### Mesh generation

The synthetic femur of the construct was meshed using MIMICs (Materialise's Interactive Medical Image Control System, Leuven, Belgium) and its other components were digitized using ABAQUS. The protocol for meshing the cortical and cancellous sections of the synthetic femur involved cubic elements with 16 nodes that were assigned to the shaft sections and tetrahedron elements with 4 nodes that were assigned to the trochanter, femoral head and methacondyles sections. The connection screws of each implant were digitized using 16-node cubic elements and the blade geometries were represented using 4-node tetrahedron elements. Standard quadratic elements were assigned to all of the remaining components of each construct.

## Results

The measurements of the biomechanical parameters of interest from the experimental tests on the synthetic femur constructs under the static and dynamic loading conditions were listed in Table 4. During the static tests, all of the constructs remained intact. LP exhibited greatest axial stiffness with a mean value of 131.4 N/mm and the corresponding values for the ABP and DCSP were 70.9 N/mm and 110.2 N/mm, respectively.

**Table 4:** Characteristics of the axial stiffness, reversible/irreversible deformation and peak load to failure following the static and dynamic loadings of the synthetic femur constructs with the implants LP, DCSP and ABP

	**ABP (n = 3)**	**DCSP (n = 3)**	**LP (n = 3)**
Axial stiffness (N/mm)			
Mean ± SD	70.9 ± 16.3	110.2 ± 12.0	131.4 ± 10.8
Total Reversible Deformation (Displacement Amplitude, mm) at 400 N			
Mean ± SD	12.4 ± 4.3	4.9 ± 1.4	4.1 ± 1.2
Total Irreversible/Plastic Deformation ((Displacement Amplitude, mm) at 1000 N			
Mean ± SD	11.3 ± 0.8	2.4 ± 0.9	1.4 ± 0.2
Peak Cyclic Load to failure (N)			
Mean ± SD	1100 ± 0	1167 ± 47.2	1600 ± 0

The results were given as mean and standard deviation (SD).

**Figure 8:**
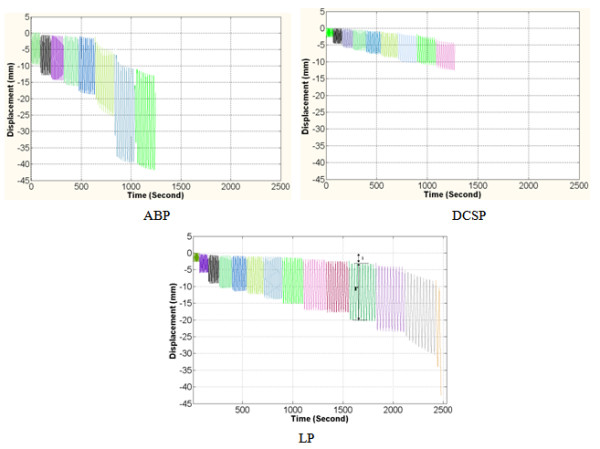
**Typical time versus displacement curves obtained from the femur constructs with implants ABP, DCSP and LP under the cyclical axial loading.** The color coding represents the steps of the incremental cyclic loading. Letters (i) and (r) represent irreversible (plastic) and reversible deformation, respectively.

The other measurements in Table 4 were associated with the dynamic axial loading of the constructs and derived from the cyclic plots similar to those shown in Figure 8. The reversible and irreversible (plastic) deformations were calculated at each increment of the dynamic loading. The overall temporal trends in the curves indicated that the amount of plastic deformation was greatest for the construct with ABP, lesser with DCSP and least with LP. When the deformation was analyzed in the linear range at the applied compression level of 400 N, the mean reversible deformation was minimal at 4.1 mm for LP, but attained slightly larger value of 4.9 mm for DCSP and reached 12.4 mm for the ABP construct. The nonlinear behaviours in response to the cyclic compression of the constructs were evaluated at a larger load of 1000 N. The mean plastic deformation was measured as 1.4 mm for LP, which was significantly lower than 11.3 mm for ABP but to a lesser degree when compared to 2.4 mm of the DCSP construct. The mean measurements of the peak load to failure indicated that the LP construct had the highest strength of 1600 N versus 1100 N for the ABP construct and 1167 N for the DCSP construct.

The above results from the static and dynamic experiments indicated that, as compared to both ABP and DCSP, the femur constructs with LP had significantly better rigidity, stability and durability, which were the important biomechanical characteristics expected of a good implant developed for fixing the fractured bone.

The hyperelastic finite element models of the implanted synthetic femurs were used to compute the vertical displacement of the top portion of the femur head where the static load was applied, the stresses induced by the plates on the proximal and distal elements of the femur and also the internal stress distributions.

The unloaded length of each femur construct was a prior knowledge used in the FE modelling but the shortened lengths after the static compression was obtained experimentally by the displacement of the stainless steel jig of the Instron machine and also estimated numerically from the FE analysis of the deformed femur construct under the specific load value for comparison. The experimental measurements and numerical estimates on the vertical displacements were listed in Table 5 for each loading step. The difference between the displacements from the experiment and the FE analysis were expressed as a percentage error and also given in the table. The small amount of error (<20 %) at each applied load indicated that the numerical models of the ABP, DCSP and LP constructs closely represented the biomechanics of the real femur construct.

**Table 5:** Comparison of the displacements measured experimentally (ED in mm) or estimated using finite element analysis (FE in mm) under static loading condition of the femur constructs

**Construct type**	**Load (N)**	**100**	**150**	**200**	**250**	**300**	**350**	**400**	**450**	**500**
ABP	ED	−3.48	−4.12	−4.82	−5.51	−6.24	−6.99	−7.77	−8.62	−9.47
	FE	−3.43	−4.03	−4.76	−5.43	−6.11	−6.81	−7.63	−8.48	−8.94
	Error	1.43 %	2.18 %	1.24 %	1.45 %	2.08 %	2.58 %	1.81 %	1.62 %	5.60 %
DCS	ED	−2.37	−3.19	−3.90	−4.41	−4.96	−5.51	−6.10	−6.71	−7.29
	FE	−2.34	−2.97	−3.76	−4.53	−5.22	−5.67	−6.31	−6.83	−7.34
	Error	1.27 %	6.90 %	3.59 %	2.65 %	4.98 %	2.82 %	3.33 %	1.76 %	0.68 %
LP	ED	−0.41	−0.72	−1.09	−1.48	−1.88	−2.31	−2.75	−3.22	−3.69
	FE	−0.34	−0.61	−0.93	−1.39	−1.93	−2.47	−2.91	−3.37	−3.81
	Error	17.07 %	15.28 %	14.69 %	6.08 %	2.59 %	6.48 %	5.50 %	4.45 %	3.15 %

Error = 100x(ED-FE)/ED.

**Figure 9:**
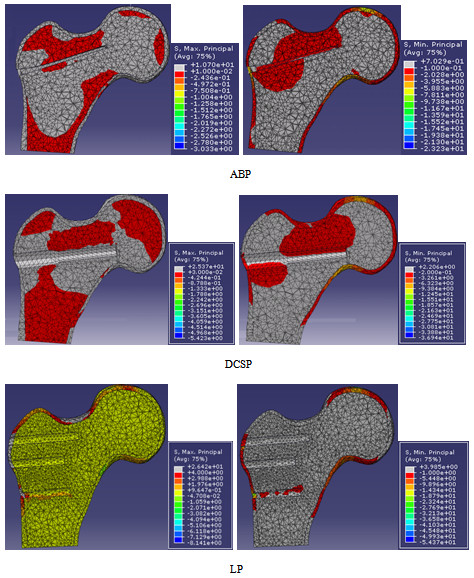
Maximum (left column) and minimum (right column) principal stress distributions in femoral heads which were implanted with ABP, DCSP and LP as viewed from the coronal midline.

**Figure 10:**
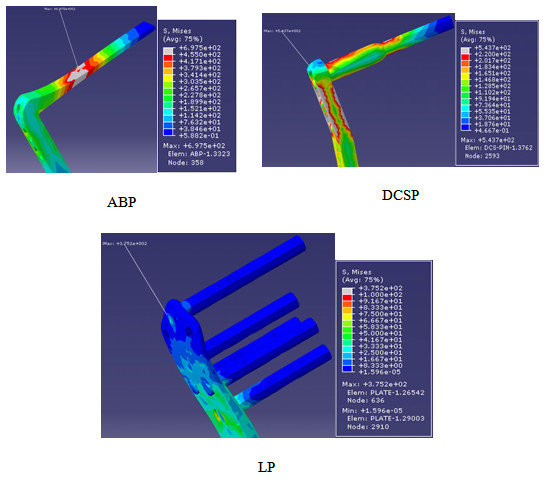
**The von Mises stress distributions in ABP, DCSP and LP.** Arrows point to the spatial location of the highest amount of von-Mises stres.

The maximum and minimum principal stresses in the femoral head were shown in Figure 9 for all of the three implant types. Table 6 compares the maximum and minimum principal stresses obtained under the maximum static load of 500 N. According to the data in Figure 9, the principal stress was spatially nonuniform for the ABP and DCSP implanted femur heads. The stress was mostly concentrated in the areas nearby the blade of ABP or condylar screw of DCSP. The overall loading pattern of the stress distribution was such that the bending action of the force applied on the top surface of the femur appeared to shear the blade and the condylar screw. This effect was more visible in Figure 10 which depicts the von Misses stress distributions in the femur head. The maximum values of the von Misses stresses were given in Table 7 along with the ranges of maximum and minimum principal stresses measured in the implants. The more uniform maximum and minimum principal stresses induced in the femur head with the LP implant implied that the load distribution was more homogeneous in the femoral head. Combining these results all together suggested that the LP implant induces lesser stress concentration and hence bone shielding in the femur than the ABP and DCSP implants.

**Table 6:** Comparison of the principal stresses induced in the femur constructs (numbers were rounded to the closest digit)

	**ABP**	**DCS**	**LP**
Maximum principal stress (MPa)	11 to −3	25 to −5	26 to −8
Minimum principal stress (MPa)	−23 to 1	−37 to 2	−54 to 4

**Table 7:** Comparison of the von Mises stresses in the three implants (numbers were rounded to the closest digit)

	**ABP**	**DCSP**	**LP**
Von Mises stress (MPa)	697	543	375
Maximum principal stress (MPa)	711 to −95	583 to −97	325 to −12
Minimum principal stress (MPa)	−766 to 450	−701 to 45	−418 to 4

## Discussion

In this study, we compared the performances of the three implants ABP, DCSP and LP in fixing SFF. Our investigation strategy involved first using a synthetic femur model, second modelling the subtrochanteric fracture by a large gap, third examining the fixations with experimental tests under both static and dynamic loading conditions, fourth performing FE analysis on the simulated constructs and finally comparing the results.

Performing the study with synthetic femur rather than cadaveric specimens improved the reproducibility of the results by reducing the intergroup variability, as such has also been reported earlier [15]. The fracture was previously represented by a large gap instead of a hair-line crack [10,16], but the analysis of this configuration using FE method was a novel approach undertaken first in the current study. The representation of a fracture with large gap made sure that the evaluation of the biomechanical performance of the construct was solely based on the characteristic response of each individual plate only, but not due to the interaction between the bone-to-bone surfaces at the proximal and distal surfaces of the fracture.

The main objective of this study was to compare the functional biomechanics of the constructs with three different orthopaedic implants. In general, orthopaedic materials are selected based on their physical and biological characteristics. Strength, flexibility, resistance to wear and corrosion and biocompatibility are the critical factors that influence the natural selection of the material. In this regard, most implants are made of metals, but with medical specifications. The implants tested in this study were manufactured using either stainless steel (ABP and DCS) or titanium alloy (LP) with grades designated by the American Society for Testing Material's standards (ASTM). Titanium offers a significantly higher strength to weight ratio than competing stainless steels. In our construct configuration, this property of titanium made the LP implant exhibit greater strength and flexibility ascompared to ABP and DCS, as indicated by the measurements in Table 4. Plate of each implant supported the load across its cross-sectional area. The transverse dimensions of the ABP and DCSP were 4.0 % and 5.9 % larger physically than that of LP, respectively. Since the working length remained nearly the same in all of the constructs (Figure 3), the material volume occupying the length of the fracture was the lowest in LP as compared to ABP and DCSP. But, the overall weights of ABP and DCSP were 57.7 % and 79.4 % heavier than that of LP. In addition, the plates made of titanium were reported to increase the cortical stiffness and bone density by 69 % and 30 %, respectively, and reduce the post surgical infection as compared to those using stainless steel [17,18]. These favourable features of LP provide the first set of evidence supporting its advantage over ABP and DCSP in fixing SFF.

Both static and dynamic lading conditions of the constructs allowed measuring the key mechanical parameters that defined the stiffness as well as the stability and durability of the constructs. Our data in Table 1 demonstrated that LP consistently exceeded in these measures compared with ABP and DCSP. As mentioned in introduction, LP offers the capability of percutaneous fixation with interlocking mechanism between the screw head and the plate. This feature stabilizes the bone fragments by means of attachment of the screw to the plate in a rigid fixed angle coupling. A fix angle device prevents excessive toggling between the screw and the plate, which in turn provides high pullout strength as well as increase in rigidity [19]. This capability makes LP a superior implant in treating unstable fractures, such as SFF. This becomes more so, when considering that the implant is required to resist large amount of compressive axial loading to provide stability for the patient mobilization. While walking, the femur is subjected to axial and bending loads as a result of the neck of femur angle or the off-set position of the femur from the trunk. This exposes the medial cortex to compressive loads while the lateral cortex is undergoing tensile loading. A stiffer implant like LP would therefore be the preferred choice during the early weight-bearing period as it reduces excessive motion at the fracture site. Consequently, potential future complications which include non-union, malunion and joint degeneration would be prevented [20].

The FE analysis demonstrated the spatial stress distributions and areas of stress concentrations in the femur heads and the implants (Figure 9). Such additional information on the biomechanical response of each construct could not be retrieved based on just the experiments. The data indicated that, in the LP implanted femur, the stress concentration on the head screws was much lower and more homogenous compared with those of ABP and DCSP. The multi-screw load carrying design on the head section of LP explains this positive outcome. With both ABP and DCSP, the exerted load was carried by the plate with only one connector device (whether the blade or condylar screw), but with LP, the load was transmitted with the help of 5 connector screws fastened into the femur head. The “multi-fastening” contour made the implanted femur construct more stable and reduced the stress concentrations by increasing the area of the load sharing or dividing the applied load through the screws [21]. From the aspect of clinical application, the lower amount of induced stress variations and concentrations in SFF fixation with LP decreases the risk factor associated with the crack development in the implanted femurs.

In this study, static loading was limited to only axial compression. Tests involving torsion of the constructs provide further information considering that this form of loading occurs in real life biological conditions. However, the use of the current loading protocol was the most appropriate since the compressive axial loading constitutes the dominant force that would be present during the partial weight bearing [22]. Therefore, in its present form, to the best of our knowledge, our results provide a baseline comparison of the biomechanical properties of the three implants using the latest experimental and numerical tools.

The use of implant-synthetic femur construct does not represent the true biological conditions where the biomechanics of the femur is influenced by the presence and attachment of the surrounding soft tissues. Numerically simulating such comprehensive model would be too complex. As in any in-vitro system, understandably, it would be particularly difficult to predict the actual clinical outcome based solely from the results of the current study when considering that a multitude of factors, including patient’s age, social habits, behaviour and smoking etc., can affect the performance of the fixation.

In the FE modelling, the CT images were acquired with a clinical scanner and had thickness of 0.6 mm, which could have been further reduced if the samples were scanned with a microCT scanner. Mesh protocol for the cortical and cancelous sections of the composite femur were completed without using special meshing tools but only the MIMICS software. Because of the complex geometry of the femur especially the head and trochanter regions, we couldn’t use cubic elements in all segments but instead used tetrahedron elements. This selection reduced the computational time. The representations of the screws in the FE modelling were simplified by using cylinder shaped pins inserted into the bone to avoid the convergence to a unique solution and also to prevent unrealistic distortion of the elements during the analysis. The bone-screw contact interfaces were defined by stiffness and tangential behaviours, as justified by the data obtained from the experimental indentation test.

The main purpose of the FE analysis was to create a numerical platform for comprehending and explaining the findings derived from the experiments. The analysis predicted the experimental data with less than 18 % error in each step of the static loading. The assignment of the hyperelastic properties to the cortical and cancellous sections of the composite femur together with the use of hierarchical framework may be the reason of such low levels of errors. Combining all these factors together, the FE models developed for the three implants in this study can potentially be further expanded and turned into a more generic form of a simulation platform for studying the performance of any other existing or future implant [23].

## Conclusion

Treating the subtrochanteric fracture of the femur is difficult and, to date, there is no one ideal implant suitable for its fixation. Our data indicate that the titanium implant LP is capable of providing more stable and durable fixation of the subtrochanteric fracture when compared to the conventional stainless steel implants ABP and DCSP. The superior biomechanical and material properties suggest that the LP implant is less likely to fail when used for fixing the subtrochanteric fractures in humans. This implant can be fixed percutaneously, and hence an extensive lateral approach as with conventional ABP and DCSP, thereby limiting periosteal striping and minimizing blood loss. Its capability of reducing non union, mal union implant failure and infection has been demonstrated in part in several clinically oriented papers, but further studies with large patient population are still warranted to rigorously demonstrate that it is a viable alternative. Also, as the understanding of the biology and biomechanics of the fractures evolve with time, new implants are likely to be designed by incorporating the acquired knowledge and performance-wise tested against those currently in use. The experimental paradigm and numerical analysis, outlined in this paper, can be used for this purpose.

## Competing interests

The authors declare that they have no competing interests.

## Authors’ Contributions

MHL performed the numerical analysis, computer tomography scans and material property measurements. KG mounted the implants to the synthetic femurs and performed the mechanical tests. SR designed the experiments and helped with the measurements. AM conceived the initial study and helped with the tests. TK contributed to the study plan and data evaluation. MB contributed to the numerical analysis, interpretation of the overall numerical and experimental results, preparation of the manuscript and its revision. All authors read and approved the final manuscript.
