# Correction: Prospects of implant with locking plate in fixation of subtrochanteric fracture: experimental demonstration of its potential benefits on synthetic femur model with supportive hierarchical nonlinear hyperelastic finite element analysis

**DOI:** 10.1186/1475-925X-11-41

**Published:** 2012-07-24

**Authors:** Mohammed Hadi Latifi, Kunalan Ganthel, Shanmugam Rukmanikanthan, Azura Mansor, Tunku Kamarul, Mehmet Bilgen

**Affiliations:** 1 National Orthopaedic Centre of Excellence in Research and Learning, University of Malaya, Kuala Lumpur, 50603, Malaysia; 2 Department of Orthopedic Surgery, Faculty of Medicine, University of Malaya, Kuala Lumpur, 50603, Malaysia; 3 Health and Translational Medicine, Faculty of Medicine, University of Malaya, Kuala Lumpur, 50603, Malaysia

## Abstract

The first publication of the work [1] did not present one of the authors' names. Kunalan Ganthel has now been added to the author list. In addition, Habib Sherkat has been added to the Acknowledgements, with thanks for providing help with the use of hyperelastic module of Abaqus Software.
